# The Antibacterial Mechanism of Baicalin and Its Solubilization Strategy

**DOI:** 10.3390/molecules31091427

**Published:** 2026-04-26

**Authors:** Chao Ning, Yuxuan Yang, Zhiyun Yu, Yantong Sun, Xin Meng, Zhiyao Dong, Haiyong Guo

**Affiliations:** 1 College of Life Science, Jilin Normal University, Siping 136000, China; 18643477064@163.com (C.N.); 15844856900@163.com (Y.Y.); yuzhiyun0808@163.com (Z.Y.); 2 School of Pharmaceutical Sciences, Jilin University, Changchun 130021, China; sunyt@jlu.edu.cn

**Keywords:** baicalin, antibacterial mechanism, solubilization technology, solubility

## Abstract

Baicalin is a natural compound sourced from *Scutellaria baicalensis* which possesses various biological activities. To date, a large amount of research has been conducted on the antibacterial activity and related mechanisms of baicalin, making it a promising candidate for new broad-spectrum antibacterial drugs. However, the solubility of baicalin is limited. To improve its solubility and overcome the clinical application bottleneck, researchers have developed various solubilization techniques. Therefore, this article introduces the biological characteristics of baicalin; explores its effects as an antibacterial agent on bacterial biofilms, quorum sensing, virulence factors, inflammatory responses, and the immune system; and discusses the applications of nano-carrier loading technology, cyclodextrin inclusion technology, metal ion coordination and organometallic complexation technology, and dynamic covalent hydrogel assembly technology in improving the solubility of baicalin, thereby enhancing its antibacterial activity.

## 1. Introduction

**Figure 1:**
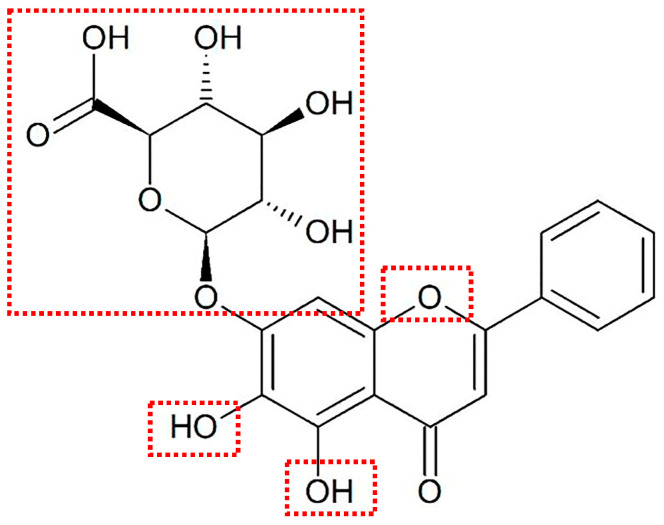
The chemical structure of baicalin. The core structural characteristics that distinguish baicalin from other medicinal flavonoids are as follows. The aglycone baicalein is linked to β-D-glucuronic acid at position 7, forming a unique 7-O-glucuronic acid glycoside, and the carboxyl sugar chain endows it with significant acidity; the A ring has a special substitution pattern of 5,6-dihydroxy (linked to sugar at position 7), which is unique to plants of the genus Scutellaria; and the 3-position of the C ring has no hydroxyl substitution, which constitutes a difference in structure and properties from flavonol compounds.

Infections caused by bacteria remain one of the major causes of human deaths worldwide. With the inappropriate use of antibiotics, especially the development of multidrug-resistant strains, researchers are seeking new types of agents to replace conventional antibiotics [1]. Many plant extracts exhibit bactericidal activity and possess complementary biological properties in anti-infection therapy, such as anti-inflammatory effects, thus emerging as key candidates for the development of antibacterial drugs [2]. Baicalin is a flavonoid compound extracted from *Scutellaria baicalensis* and represents one of the principal active components of this medicinal plant [3]. Its chemical structure is formed by the conjugation of the C7 hydroxyl group of baicalein with glucuronic acid (Figure 1). Its appearance is in the form of a pale yellow powder, with a bitter taste. It exhibits good solubility in organic solvents such as chloroform, nitrobenzene, and dimethyl sulfoxide, and is sparingly soluble in water and alcohol-based solvents [3,4]. This compound exhibits diverse biological activities, such as antibacterial [5], antioxidant [6], anti-cancer [7], and anti-inflammatory effects [8], and is therefore widely used in the research of drug formulations. Accumulating evidence has confirmed that baicalin can effectively treat various bacterial infections including pneumonia [9], meningitis [10], gastrointestinal infections [11], and sepsis [12]. However, baicalin has extremely low water solubility and permeability, with an absolute bioavailability after oral administration of only 2.2% [13], which poses great challenges to its clinical translation. Therefore, this review elucidates the biological characteristics and antibacterial mechanism of baicalin, summarizes the current solubilization technologies for improving its solubility, and provides a theoretical basis for its clinical application in combating bacterial infections.

## 2. The Biological Characteristics of Baicalin

### 2.1. Selective Toxicity

The selective toxicity of baicalin is based on the biological differences between tumor cells and normal cells, aiming to interfere with the survival and proliferation of tumor cells without causing damage to normal cells (Table 1). Studies have found that baicalin has no toxicity to osteoblast MC3T3-E1 and human bone marrow mesenchymal stem cells hMSC, but significantly reduces the viability of human breast cancer cells MDA-MB-231 and MCF-7 [14]. Another study found that a low concentration of baicalin (0.625 μM) treated alone had no obvious toxicity to human normal liver cells QSG-7701 and mouse primary liver cells, and there was no significant decrease in cell viability, while it had a significant inhibitory effect on human liver cancer cells MHCC-97H and HCC-LM3 [15]. At the tissue level, Yang et al. [16] used a patient-derived xenograft (PDX) derived from kidney cancer patients to study the effect of baicalin in the cardiotoxicity induced by sunitinib. The results showed that baicalin (100 mg/kg/d) used alone had no obvious cardiotoxicity, hepatotoxicity, or nephrotoxicity, and had no adverse effect on mouse body weight. When combined with sunitinib treatment, it could significantly alleviate the cardiotoxicity induced by sunitinib and did not weaken its anti-tumor effect [16]. In addition, baicalin was also found to be non-toxic to normal cells such as human liver cells L02 [17] and human embryonic lung fibroblasts WI-38 [18], and had certain protective effects on normal cells or tissues damaged by drugs, radiation, etc. Cisplatin can inhibit the activity of normal canine renal epithelial cells (MDCK); promote significant increases in the levels of nitric oxide, malondialdehyde, and inflammatory factors TNF-α, IL-1β in MDCK cells; and significantly decrease the activities of superoxide dismutase, glutathione peroxidase, and catalase [19]. However, baicalin treatment led to opposite changes in these factors, alleviating the damage of cisplatin to MDCK cells [19]. In addition, Wang et al. [20]’s study found that baicalin (25–200 μg/mL) used alone had no toxicity to normal human skin epidermal cells HaCaT, and could dose-dependently reverse the decrease in cell viability, increase in apoptosis, excessive generation of ROS, and formation of cyclobutane pyrimidine dimers and oxidative adducts caused by UVC radiation in normal human skin epidermal cells HaCaT.

### 2.2. Stability

The degradation of baicalin is dependent on pH and temperature. Feng et al. [21] found that the monomer of baicalin was stable at pH 2.0–4.5 for 24 h, and the half-life at pH 6.8, 7.4, 9.0 (25 °C) was 2.89 h, 1.81 h, 0.87 h, respectively. At 4 °C, the half-life reached 71.7 h, and vitamin C could completely stabilize it, while sodium sulfite could extend its half-life from 1.81 h to 3.45 h [21]. At pH 6.8, almost no degradation occurred in the total flavonoids part of the *Scutellaria baicalensis*, and the half-life at pH 7.4 was extended from 1.81 h to 2.87 h. After protection by acidification, low temperature, and addition of antioxidants, the total flavonoids part of *Scutellaria baicalensis* in plasma, urine, and liver, stomach, intestine, and kidney tissue homogenates could be prepared and stored for 24 h, 3 freeze–thaw cycles, and −20 °C for about 15 days. Xing et al. [22] obtained similar results. They used LC/MS^n^ and electron paramagnetic resonance spectrometry (EPR) techniques to find that the half-life of baicalin in 25 °C, pH 7.4 buffer solution was 1.75 h, while that in pH 9.0 buffer solution was only 0.83 h. The half-life would be accelerated with temperature increase; at 35 °C and 45 °C, it was further shortened to 0.52 h, 0.16 h (pH 7.4) and 0.30 h, 0.15 h (pH 9.0) [22]. In biological matrices, the half-life of baicalin in plasma at 25 °C was 2.30 h, in urine it was 0.81 h, and in liver homogenate it was only 0.14 h. The main degradation process of baicalin in vitro in plasma and urine is mediated by phenoxyl radical redox reaction, while when added to tissue homogenates, baicalin mainly undergoes hydrolysis and phase II metabolic pathways. This study also found that acidification (pH 3.0–4.0) could stabilize baicalin in urine and plasma [22]. The combination of baicalin with stabilizers to prepare baicalin nanocrystals has been found to improve the stability of baicalin. Xie et al. [23] used nanocrystalline cellulose–methylcellulose sodium (NCCS) as a synergistic stabilizer and combined with spray drying technology to prepare baicalin nanocrystals, which had good physical stability and could be stored stably at 4 °C and 25 °C for 6 months. Jin et al. [24] prepared baicalin microcrystals and significantly improved the physical stability of baicalin, with no significant changes in particle size, potential, and dispersion after storage at room temperature for 90 days.

### 2.3. Low Resistance

Baicalin can reduce bacterial resistance through both direct and indirect killing pathways. In the direct killing mechanism, baicalin can eliminate the resistance mediated by the biofilm of highly virulent *Klebsiella pneumoniae* (*K. pneumoniae*) by down-regulating the expression of key genes *AcrA* and *wbbM* in the biofilm [25]; it can also reduce the drug resistance of *Helicobacter pylori* (*H. pylori*) by binding to the multidrug efflux pump protein MexB and down-regulating the expression of multidrug resistance genes *hefA* and virulence gene *vacA* [26]. Furthermore, it can enrich oxidative phosphorylation, biofilm regulatory system, and trehalose synthesis pathways to directly reduce the drug resistance and colonization survival ability of *Acinetobacter lwoffii* (*A. lwoffii*) [27]. Baicalin has also been found to have the potential to reverse antibiotic resistance; for example, baicalin significantly reduced the resistance of methicillin-resistant *Staphylococcus aureus* (MRSA) and *Stenotrophomonas maltophilia* (*S. maltophilia*) to ampicillin by disrupting the integrity of the bacterial cell wall and cell membrane, inducing excessive ROS generation, and inhibiting the expression of multiple multidrug efflux pump genes (such as *oprM*, *adeA*, and *acrB*) [28]. Baicalin can also directly reverse plasmid-mediated polymyxin resistance by disrupting the integrity of the bacterial outer membrane, enhancing cell membrane permeability, and neutralizing the charge changes related to resistance [29]. In the indirect killing mechanism, baicalin can regulate the immune response during *Staphylococcus aureus* (*S. aureus*) infection by improving mitochondrial function and dynamics, reducing inflammatory responses in vivo and in vitro [5]. Therefore, it does not induce resistance gene mutations, does not generate resistance selection pressure, and can independently or replace antibiotics to control drug-resistant bacterial infections. Moreover, baicalin can also weaken the resistance of tumor cells to anti-cancer drugs. The resistance to DDP (cisplatin) in lung cancer has been widely reported. Xu et al. [30] found that the MARK2 mRNA level and the protein expressions of MARK2 and p-Akt in human lung cancer A549/DDP cells (cisplatin-resistant strain) were significantly higher. Baicalin can dose-dependently reduce the mRNA and protein expressions of MARK2 and MARK2 and p-Akt in A549/DDP cells, thereby reducing the resistance of A549/DDP cells to DDP and achieving a synergistic anti-cancer effect when used in combination with cisplatin [30]. Wu et al. [31] discovered the mechanism by which baicalin reverses the resistance of HNSCC cells to cisplatin; namely, baicalin can inhibit glutathione S-transferase (GST)-π subtype (also known as GSTP1) activity by interacting with the Cys47 residue of GSTP1, thereby enhancing the accumulation of cisplatin in cells or promoting activation of the JNK pathway. In addition, baicalin has been found to reverse the resistance of non-small cell lung cancer to PD-1 through regulating the levels of metabolites of the intestinal microbiota, such as butyric acid, propionic acid, and valeric acid, optimizing the ratio of PD-1^+^CD8^+^ T cells/PD-1^+^ Treg in the tumor microenvironment and, thereby, reversing the resistance to PD-1 in non-small cell lung cancer [32].

### 2.4. Pharmacokinetics

The in vivo pharmacokinetic behavior of baicalin exhibits typical characteristics of rapid absorption, significant exposure differences, metabolic dependence on intestinal flora, and slow elimination [33]. Both oral and intraperitoneal administration of baicalin can be rapidly absorbed, and the drug can be detected in the plasma of rats 15 min after administration [33]. The peak time (Tmax) is 0.25 h, indicating its kinetic advantage of rapid entry into the bloodstream [34]. The peak concentration (Cmax) after intraperitoneal injection is 24.9 ± 2.93 μg/mL, and the area under the curve (AUClast) is 32.7 ± 6.2 μg · h/mL; while the Cmax after oral administration is only 1.87 ± 0.45 μg/mL, and the AUClast is 17.9 ± 3.4 μg · h/mL [33]. The exposure after intraperitoneal injection is significantly higher than that after oral administration. In addition, baicalin shows a typical double-peak blood concentration–time curve in vivo, with a second peak appearing 6–12 h after administration [33]. This feature is mainly mediated by intestinal circulation, and the overall elimination rate is slow, allowing for prolonged effective exposure in the body.

The intestinal microecology is the core determinant factor regulating the oral absorption, metabolic transformation, and pharmacokinetic characteristics of baicalin. Xing et al. confirmed that after oral administration of baicalin in normal rats, a typical double-peak drug–time curve was presented, with significant intestinal circulation characteristics [34]. The main pharmacokinetic parameters were: Tmax = 6.2 ± 1.8 h, Cmax = 1733.6 ± 756.1 ng/mL, AUC_0_→∞ = 22,460.3 ± 9621.9 ng · h/mL, and t_1_/_2_ = 9.6 ± 1.7 h [34]. Its oral absorption is highly dependent on the activation mediated by intestinal flora hydrolysis. After baicalin enters the intestine, it needs to be hydrolyzed by the β-glucosidase produced by intestinal flora into baicalein, and then undergoes glucuronidation, methylation and other phase II conjugation reactions in the intestine and liver, generating metabolites such as baicalein monoglucuronide and luteolin A, thus completing the activation and absorption process [34]. After pre-treatment with antibiotics to clear the intestinal flora, the hydrolysis activation, absorption and metabolism of baicalin were severely inhibited. The Tmax was significantly advanced to 0.3 ± 0.1 h, Cmax was significantly reduced to 777.3 ± 69.2 ng/mL, the AUC was significantly reduced to 6115.4 ± 2893.3 ng · h/mL, and the t_1_/_2_ was significantly prolonged to 17.8 ± 1.7 h [34]. Thus, the overall oral absorption efficiency and bioavailability sharply decreased [34]. Therefore, the intestinal flora mediates the activation, transmembrane absorption and in vivo pharmacokinetic characteristics of baicalin. Most baicalin must rely on the activation by the intestinal flora to be effectively absorbed, the direct passive absorption efficiency is extremely low, and the oral bioavailability is low.

### 2.5. High Target Specificity

Baicalin has a specific effect of targeting and regulating the intestinal microecology. It can inhibit the colonization and virulence of pathogenic bacteria without adverse effects on beneficial bacteria, and can precisely restore the disrupted intestinal flora structure. Peng et al. [35] confirmed that the pretreatment with baicalin (200 mg/kg) could significantly reduce the colonization of avian pathogenic *Escherichia coli* (APEC) in the lungs; reduce the release of pro-inflammatory factors such as TNF-α, IL-1β, and IL-6; and repair damage to the blood–air barrier by restoring the expression of tight junction proteins such as ZO-1, occludin, and claudin-3 [35]. Further mechanisms indicated that baicalin could reshape the intestinal flora disorder induced by APEC, significantly increase the abundance of short-chain fatty acid (SCFA)-producing bacteria such as *Blautia*, *Lachnospiraceae*, and *Intestinimonas* [35]. Zhang et al. [36] found that baicalin (100 mg/kg) could directly inhibit the TLR4/IRF/STAT signaling pathway, reduce the polarization of pro-inflammatory M1-type macrophages, and restore the Th17/Treg balance to alleviate intestinal inflammation and tissue damage [36]. At the same time, it could significantly enrich *Lactobacillus amylovorus* in the intestine, thereby reducing the intestinal inflammatory response caused by *Escherichia coli* (*E.coli*) [36]. In addition, baicalin can specifically reverse the intestinal flora imbalance caused by combined antibiotics, increase the abundance of beneficial bacteria such as *Lachnospiraceae* and *Akkermansia*, and enhance the intestinal resistance to *Clostridium difficile* (*C. difficile*) colonization [37]. In summary, baicalin exerts specific anti-infection and intestinal protection effects by selectively inhibiting the colonization and virulence of pathogenic bacteria, protecting and promoting the proliferation of beneficial bacteria, precisely restoring the imbalance of the flora, and enhancing intestinal resistance to *C. difficile* colonization.

## 3. The Antibacterial Mechanism of Baicalin

**Figure 2:**
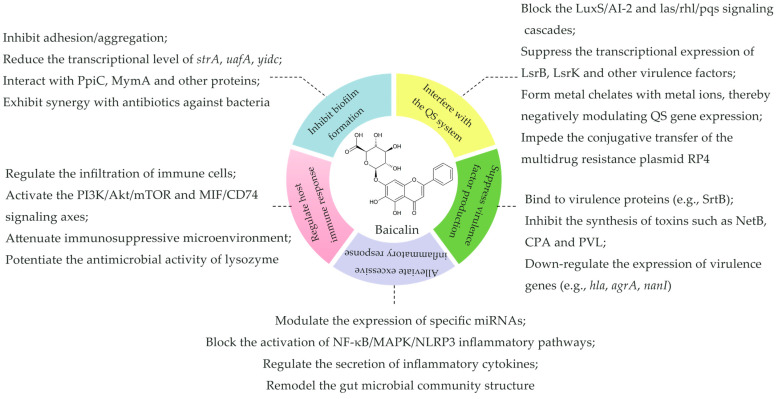
The inhibitory mechanism of baicalin on bacterial colonization and drug resistance. The mechanism of baicalin on bacteria includes five core pathways: inhibiting biofilm formation, interfering with the quorum sensing (QS) system, suppressing the production of virulence factors, regulating the host immune response, and alleviating excessive inflammatory responses. Through blocking signal pathways, regulating gene expression, regulating the infiltration of immune cells and reshaping the intestinal microbiota, it exerts pharmacological activities such as antibacterial, anti-inflammatory and immunomodulatory effects.

Unlike traditional antibiotics, which primarily act by directly killing bacteria or inhibiting their proliferation, plant extracts—endowed with diverse natural origins, broad-spectrum antibacterial activity, and unique mechanisms of action—can exert antibacterial effects without imposing strong selective pressure on bacteria. This makes them far less likely to induce the emergence of drug-resistant strains, positioning plant extracts as a pivotal research direction for replacing or supplementing traditional antibiotics [38,39,40,41]. Baicalin, as the core bioactive flavonoid isolated and purified from the medicinal botanical *Scutellaria baicalensis*, has been extensively validated for its antibacterial potential. It exhibits potent inhibitory effects against a variety of clinically prevalent infectious microorganisms, including (but not limited to) *S. aureus* [42], *E. coli* [42], *S. maltophilia* [28], and *H. pylori* [26]. A systematic review of existing research reveals that baicalin’s antibacterial activity does not depend on a single target but is mediated through a multi-pathway, multi-dimensional regulatory network (Figure 2). Specifically, it can: (i) disrupt the formation and stability of bacterial biofilms; (ii) interfere with the group sensing (QS) system; (iii) suppress the synthesis of virulence factors; (iv) modulate host inflammatory signaling pathways to mitigate tissue damage caused by pathogens; and (v) regulate host immune function to enhance the body’s capacity for pathogen clearance (Figure 2). These multi-targeted and synergistic characteristics not only endow baicalin with potent antibacterial activity, but also confer it substantial advantages in circumventing bacterial resistance. Collectively, these findings provide a critical theoretical foundation for the development of new antimicrobial agents, offering promising strategies to address the increasingly severe problem of antibiotic-resistant bacterial infections.

### 3.1. The Effect on Biofilms

Biofilms are structured communities formed by bacteria to adapt to their environment. Composed of bacterial cells and their self-secreted extracellular polymers (mainly including polysaccharides, proteins, and extracellular DNA), they can firmly adhere to various surfaces and serve as an important survival form for bacteria [43]. Biofilms can protect pathogenic bacteria from being eliminated by the host’s immune system, hinder the action and penetration of antibiotics, thereby promoting the progression of chronic infections and inducing persistent infections related to drug resistance [44,45]. Therefore, it is of great significance to develop and screen new anti-biofilm molecules that can effectively intervene and eliminate infections related to biofilms.

A large number of studies have confirmed that baicalin has antibacterial biofilm activity against bacteria and is expected to become a highly promising anti-biofilm agent. Biyik et al. [46] found that baicalin inhibited biofilm formation in 7 strong biofilm-producing strains (*Pseudomonas aeruginosa*, *P. aeruginosa*) at sub-minimum inhibitory concentration (sub-MIC), with an inhibition rate of 67.00–90.64%. *Staphylococcus saprophyticus* (*S. saprophyticus*) also frequently causes various intractable infections due to biofilm formation. The research conducted by Wang et al. [47] revealed that baicalin inhibited the adhesion and aggregation of *S. saprophyticus* biofilms by down-regulating the mRNA transcription levels of key functional genes (*srtA*, *uafA*, *Aas*, *lytM*, *ssp*) involved in bacterial surface structure assembly, adhesion colonization, and biofilm formation. Additionally, Ma et al. [27] demonstrated that baicalin exhibited bactericidal activity against *A. lwoffii* biofilms isolated from milk. In particular, 4 minimum inhibitory concentration (4 MIC) of baicalin reduced the viable cell count in *A. lwoffii* biofilms by 0.67 log10 CFU/mL [27]. After baicalin treatment, the trehalose content in *A. lwoffii* biofilms decreased significantly; the expression of genes *yidC*, *rpiJ*, and *liuE* (involved in biofilm formation) was downregulated, while the expression levels of genes *otsA*, *betB*, *mprA*, *rcsC-3*, *otsB*, and *ahpC* (related to oxidative phosphorylation and two-component systems (TCS)) were significantly upregulated [27]. Molecular docking analysis uncovered that baicalin has good binding potency toward biofilm-related target proteins (e.g., ppiC, mymA, mprA) of *A. lwoffii*, suggesting that it may exert anti-biofilm effects by regulating these target proteins [27].

Baicalin with antibiotics or other plant extracts can also enhance the anti-biofilm activity of antibiotics. Highly pathogenic *K. pneumoniae* has a strong ability to form biofilms. When baicalin is used in combination with levofloxacin, it can disrupt the three-dimensional structure of early *K. pneumoniae* biofilms, reduce early biofilm biomass and bacterial load, and downregulate the expression level of genes *AcrA* (periplasmic adaptor protein of the efflux pump AcrAB-TolC) and *wbbM* (participation in the biosynthesis of the O-antigen of lipopolysaccharide) [48]. This clearly indicates that the combination may inhibit *K. pneumoniae* biofilm formation by regulating the expression of efflux pump and lipopolysaccharide biosynthesis-related genes [48]. The WalK/R (also known as YycG/YycF) two-component system is a key regulatory system for the metabolism of the cell wall in *staphylococci* [49]. Most of the cell wall anchoring proteins are also the core components of the matrix of the multidrug-resistant (MDR) *S. saprophyticus* biofilm [50]. Relevant studies have found that the combination of baicalin with azithromycin reduced the minimum inhibitory concentration (MIC) of azithromycin against MDR *S. saprophyticus* biofilms by 4–512 fold [51]. Compared with the group treated with the drug alone, the combination of azithromycin and baicalin decreased the dispersion rate of MDR *S. saprophyticus* biofilms [51]. Furthermore, after treatment with baicalin alone or in combination with azithromycin, compared with the azithromycin-alone treatment group and the blank control group, the transcriptional levels of the MDR *S. saprophyticus* WalK/R system-related genes *WalK*, *WalR*, *yycI*, and *yycH* were all upregulated [51], indicating that baicalin can enhance the bactericidal effect of azithromycin against MDR *S. saprophyticus* biofilms by regulating the WalK/R system. Additionally, the combination of baicalin with carvacrol exhibited a significant synergistic effect against *Salmonella Typhimurium* (*S. Typhimurium*) on food and food contact surfaces, significantly enhancing the disruption of *S. Typhimurium* biofilm structure and greatly reducing bacterial viability [52].

### 3.2. The Effect on Quorum Sensing

Quorum sensing (QS) represents a complex bacterial cell–cell communication network. Bacteria can produce and detect QS signaling molecules, thereby sensing the composition and density of the microbial community in their complex ecological niche, and dynamically regulating their own gene expression to adapt to environmental changes [53]. Numerous pathogenic bacteria depend on QS to modulate the acquisition and expression of resistance traits. Therefore, targeting QS has been proven to be an effective alternative strategy for controlling infections compared to traditional antibiotics [54].

**Figure 3:**
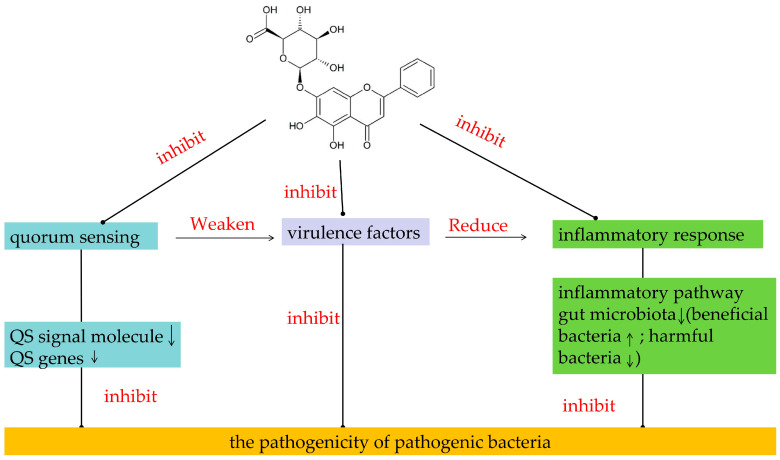
Mechanism of antibacterial and anti-inflammatory effects of baicalin. Baicalin inhibits QS to indirectly down-regulate virulence factors, reducing the damage to host cells caused by bacteria (such as mucosal injury and tissue inflammation), and simultaneously reducing the ability of bacteria to induce excessive inflammatory responses in the host.

A wealth of studies has shown that baicalin exerts antibacterial effects by targeting the QS signaling pathway and regulating the expression of related genes (Figure 3). For instance, baicalin suppresses the LuxS/AI-2 QS system in extraintestinal pathogenic *E. coli* (ExPEC), thereby attenuating the expression of the *luxS*. This inhibition reduces the biosynthesis of the signaling molecule AI-2, and consequently inhibits ExPEC biofilm formation and self-aggregation [55]. In a mouse peritoneal implantation model of *P. aeruginosa* infection, baicalin downregulated the expression levels of QS genes such as *lasI*, *lasR*, *rhlI*, *rhlR*, *pqsR* and *pqsA*, thereby significantly reducing the concentrations of 3-oxo-C12-HSL and C4-HSL (QS signaling molecules), and also enhancing the clearance effect of *P. aeruginosa* in vivo [56]. Peng et al. [57] demonstrated that baicalin significantly abrogates QS by reducing the expression levels of virulence genes (e.g., *LsrB*, *LsrK*) in avian pathogenic *Escherichia coli* (APEC), thereby attenuating APEC pathogenicity. Additionally, Wang et al. [58] also discovered that baicalin inhibits the excretion pump MsrA of *S. saprophyticus*; down-regulates the expression of the quorum sensing genes *agrA*, *agrC*, *RNAIII* and *sarA*; and inhibits the formation of biofilms.

Notably, metal complexes formed by baicalin and metal ions exhibit targeted activity against bacterial QS systems [59]; for example, baicalin-aluminum significantly downregulates the mRNA expression levels of *agrB* (encoding an autoinducing peptide [AIP] processing and transport protein) and *agrD* (encoding an AIP precursor peptide) in the Agr QS system of *Clostridium perfringens*, as well as *virS* (encoding a membrane sensor histidine kinase) and *virR* (encoding a response regulator) in the VirS/R two-component regulatory system [59]. Therefore, baicalin-aluminum may indirectly inhibit biofilm formation by interfering with the Agr QS system [59]. Furthermore, antibiotic resistance genes (ARGs) spread rapidly among bacteria through conjugation, significantly accelerating the process of drug resistance transmission. Baicalin binds to LsrB, thereby modulating QS system activity and inhibiting the conjugative transfer of the multidrug resistance plasmid RP4 [60].

### 3.3. The Effect on Virulence Factors

Virulence refers to the potential ability of a pathogen to invade a host and cause disease, while virulence factors are the important molecules that facilitate the colonization of bacteria in host cells [61]. These virulence factors are essentially cytoplasmic, membrane-associated, or secreted. Cytoplasmic virulence factors can facilitate bacteria to undergo rapid adaptive metabolic adjustments as well as physiological and morphological changes. Membrane-associated virulence factors assist bacteria in adhering to and evading host cells [61]. The secreted virulence factors not only enable bacteria to evade the host’s immune response, but also can kill host cells through a synergistic effect [61,62].

*Porphyromonas gingivalis* (*P. gingivalis*) lipopolysaccharide (LPS) is an essential virulence factor promoting periodontal disease. Luo et al. [63] found that baicalin may down-regulate the expression levels of interleukin-6 (IL-6) and interleukin-8 (IL-8) in human oral keratinocytes induced by *P. gingivalis* LPS through negative regulation of Toll-like receptor (TLR) signaling, while simultaneously inhibiting the expression of Nuclear factor kappa-light-chain-enhancer of activated B cells (NF-κB), p38 mitogen-activated protein kinase (p38 MAPK), and c-Jun N-terminal kinase (JNK) activated by *P. gingivalis* LPS [63].

Sortase B (SrtB) is a cell surface-anchored transpeptidase in *S. aureus*. Baicalin can specifically bind to the amino acid residues Asn^92^ and Tyr^128^ of SrtB, inhibiting the activity of SrtB and reducing the pathogenicity of *S. aureus* [64]. Additionally, baicalin can control the virulence of MRSA by inhibiting the expression of virulence-related factors *hla* and *argA* [65].

*Streptococcus agalactiae* (*S. agalactiae*), as the main pathogen of fish, can cause high incidence and mortality rates in various farmed fish species, resulting in serious aquaculture diseases [66]. Hemolysin/cytolysin and hyaluronidase are key virulence factors of *S. agalactiae* [67,68]. Recent studies have shown that baicalin can significantly inhibit the activity or synthesis of these two virulence factors (Figure 3) [69]. Notably, 4 μg/mL baicalin completely inhibited hyaluronidase production and reduced the β-hemolytic/cytolytic activity of *S. agalactiae* by 22.11% to 84.54%. In vivo studies further demonstrated that baicalin significantly inhibited the mortality rate of *S. agalactiae*-infected tilapia, with a median effective concentration (EC50) of 525.8 mg/kg [69].

As a secreted pore-forming toxin of *S. aureus*, Panton-Valentine leukocidin (PVL) plays a crucial regulatory role in the pathogenesis of mastitis [70]. Hou et al. [71] found that pre-treatment with baicalin can significantly alleviate the G0/G1 phase arrest of bovine mammary epithelial cells (BMECs) caused by recombinant PVL (rPVL), and promote the transition of cells to the S phase and G2/M phase. In vivo experiments, the breast tissue structure of mice was disrupted, with inflammatory cell infiltration (neutrophils and lymphocytes) in the mammary duct lumen and thickening and edema of the mammary duct wall in the *S. aureus* ATCC49775 (PVL-producing strain) or rPVL treatment groups [71]. When pretreated with 100 mg/kg baicalin, the mammary lobule structure of mice was intact, the alveoli were not damaged, and only a small amount of inflammatory cells infiltrated locally, with significant reduction in pathological damage [71]. Moreover, this study also found that baicalin can specifically increase the phosphorylation levels of cell cycle-related proteins, such as BCLAF1 (S285), CDK7 (T170), NF2 (S518), and PKM2 (S37) [71]. These findings demonstrate that baicalin can alleviate the pathological damage of mammary tissue induced by *S. aureus* and rPVL, and its mechanism is tightly associated with the regulation of phosphorylation of cell cycle-related proteins and the preservation of normal cell proliferation cycle.

### 3.4. The Effect on the Inflammatory Response

Baicalin can exert anti-inflammatory effects on diseases related to bacteria through regulating the expression of microRNAs, inhibiting inflammatory signaling pathways, and modulating the intestinal flora (Figure 3). Porcine *Haemophilus parasuis* (*H. parasuis*) is the pathogen causing Glässer’s disease. Its infection process can lead to vascular damage in pigs, and it is directly related to the occurrence of vascular inflammation [72]. The research conducted by Fu et al. [73] revealed that the expression level of microRNAs is closely related to the inflammatory damage of vascular tissues caused by infection with porcine *H. parasuis*. Baicalin can significantly reverse the abnormal expression of microRNAs induced by *H. parasuis* infection, such as significantly down-regulating the overexpressed ssc-miR-375 [73]. The microRNA target genes regulated by it are enriched in peroxisome, oxidative phosphorylation and other related pathways. This indicates that baicalin may alleviate the vascular inflammation and injury caused by Glässer’s disease through activating antioxidant and DNA repair-related pathways, enhancing cellular defense capabilities [73].

Sepsis is a life-threatening disease characterized by an imbalanced immune response of the host to an infection, leading to organ dysfunction [74]. In a mouse model of sepsis, baicalin was found to exert anti-inflammatory effects by targeting multiple inflammatory signaling pathways, such as extracellular regulated kinase (ERK), JNK MAPK and NF-κB pathways [75]. It inhibited the production of IL-6, tumor necrosis factor-α (TNF-α) and other cytokines by Mφs and dendritic cells (DCs) stimulated by MRSA or bacterial mimics, thereby protecting the mice from methicillin-resistant *S. aureus* infection [75]. In addition, the researchers also investigated whether baicalin could effectively alleviate the inflammatory response induced by ExPEC in 3D4/21 cells [76]. The results showed that baicalin could significantly down-regulate the expression levels of pro-inflammatory cytokines in 3D4/21 cells after pig ExPEC infection, including interleukin 1β (IL-1β), interleukin 6 (IL-6), and interleukin 8 (IL-8) [76]. At the same time, baicalin could significantly inhibit the phosphorylation processes of proteins such as P65, nuclear factor κB inhibitor α (IκBα), extracellular regulatory kinase (ERK), c-Jun amino-terminal kinase (JNK), and P38, as well as reducing the expression levels of NLRP3 inflammasome, apoptosis-associated speck-like protein (ASC), and caspase-1 protein [76]. These results indicate that baicalin can effectively alleviate the damage caused by pig ExPEC infection to 3D4/21 cells by inhibiting the activation of the NF-κB/MAPK signaling pathway and blocking the activation process of the NLRP3 inflammasome.

One of the important causes of intestinal inflammatory damage in weaned piglets is stimulation by bacterial lipopolysaccharide (LPS) [77]. Baicalin can alleviate LPS-induced inflammatory responses by regulating multiple inflammatory signaling pathways; for example, baicalin can reduce LPS-induced intestinal epithelial cell inflammation in pigs by inhibiting PARP1-mediated NF-κB and NLRP3 signaling pathways [78]. It can also protect against LPS-associated acute lung injury [79] and periodontitis [80] by activating the Nrf2/ARE or PI3K/AKT/mTOR signaling pathways, respectively. Furthermore, baicalin not only exhibits significant anti-inflammatory activity, but also can moderately regulate the intestinal flora. Li et al. [81] found that baicalin exhibited anti-inflammatory effects in a rat model of acute pneumonia induced by multidrug-resistant (MDR) *P. aeruginosa*. Pyrosequencing of 16S rRNA genes in rat feces revealed that baicalin effectively increased the abundance of beneficial intestinal symbiotic bacteria (*Ligilactobacillus*, *Lactobacillus* and *Bacteroides*), but decreased the abundance of harmful intestinal symbiotic bacteria (*Muribaculaceae* and *Alistipes*) [81].

### 3.5. The Effect on the Immune Response

Baicalin can exert an immunoregulatory effect by directly regulating the quantity and function of immune cells. The research conducted by Zhu et al. [82] revealed that baicalin reduced the number of neutrophils in the lungs, livers, and peritoneal lavage fluid of mice with multi-microbial sepsis. Compared with the cecal ligation and puncture group, the apoptosis rate of thymus CD3^+^ T cells in the baicalin group was decreased, while the number of spleen CD4^+^ and CD8^+^ T lymphocytes and dendritic cells (DC) was increased, and the number of CD4^+^CD25^+^ regulatory T cells was decreased. This indicates that baicalin can improve the survival rate of mice with polymicrobial sepsis by inhibiting lymphocyte apoptosis [82]. Subsequently, Xue et al. [83] investigated the intervention effect of baicalin on the *Pneumocystis* pneumonia model in immunosuppressed rats. The study found that compared with the trimethoprim-sulfamethoxazole group, rats treated with baicalin had more CD4^+^ T cells and fewer CD8^+^ T cells, and the inflammatory cytokine profile was weakened [83]. Additionally, it has been found that baicalin can enhance the clearance ability of macrophages against *S. aureus* by improving mitochondrial function and dynamics, directly strengthening the antibacterial immune response [5].

Baicalin can also alleviate immunosuppression by targeting key immune signaling pathways, regulating cytokines, and activating the autophagy pathway. *Glaesserella parasuis* (*G. parasuis*, synonym *H. parasuis*) is involved in the early colonization process of microorganisms in piglets’ nasal cavities [84] and can cause immunosuppression in piglets. Fu et al. [85] found that baicalin reduced the expression of IL-1β, IL-6, IL-8, IL-18, TNF-α, and COX-2 in the spleen of piglets through the polarization of the MIF/CD74 signaling pathway and increased the level of CD74 protein in the spleen of piglets induced by *G. parasuis*. The study also found that baicalin regulated the activation of the PI3K/Akt/mTOR and the RAF/MEK/ERK signaling pathway; changed the levels of autophagy-related proteins (e.g., Beclin-1, P62, and LC3B); promoted the polarization from M2 to M1; and increased the levels of CD3, CD4, CD8, and TIM3 in the spleen of piglets, thereby alleviating the immunosuppression triggered by *G. parasuis* in piglets [85].

The initiation of the PD-1/PD-L1 axis can induce host immune suppression [86]. Therefore, researchers evaluated whether baicalin could alleviate the immunosuppression induced by *G. parasuis* in piglets by repressing the activation of PD-1/PD-L1. They discovered that baicalin inhibited the PI3K/Akt/mTOR and RAS/MEK/ERK signaling pathways, thereby down-regulating the transcription of pro-inflammatory factors and immune regulatory factors such as *IL-1β*, *IL-10*, *IL-18*, *TNF-α* and *IFN-γ* in the blood, and up-regulating the transcription of immune activation-related factors *IL-2* and *IL-8* in the blood [87]. Further studies found that baicalin could increase the proportion of CD3^+^ T cells, CD3^+^CD4^+^ T cells, CD3^+^CD8^+^ T cells, and CD3^−^CD21^+^ B cells in the spleen cell population, as well as the proportion of CD3^+^ T cells, CD3^+^CD4^+^ T cells, and CD3^+^CD8^+^ T cells in the blood, while inhibiting the activation of PD-1/PD-L1 and TIM-3 [87]. In addition, baicalin has also been found to suppress the protein expression of p-PI3K, p-Akt, and p-mTOR, as well as the ratio of p-MEK1/2/MEK1/2 and p-ERK1/2/ERK1/2, and increase the protein expression of RAS [87], demonstrating that baicalin can mitigate the immunosuppression in piglets mediated by the PD-1/PD-L1 axis infected with *G. parasuis*. Furthermore, baicalin can regulate CD163/TWEAK axis, inhibit the activation of Notch/Wnt and NLRP3/Caspase 1 signaling pathways, and promote autophagy, so as to inhibit the production of cytokines in blood vessels of piglets challenged with *G. parasuis*, and reduce their pathological damage [88]. Researchers have also found that baicalin can exert a stronger antibacterial effect in the innate immune response of bacteria by binding to enzymes. Lysozyme, as an important component of the humoral immunity of the innate immune system, plays a central role in the host’s defense response against pathogen infections [89]. Gao et al. [90] used a mouse model of *S. aureus* mastitis and a model of neutrophils infected with *S. aureus* to test the effect of baicalin on lysozyme activity. The findings demonstrated that *S. aureus* infection significantly upregulated the mRNA and protein expression of lysozyme in mammary tissue. When exposed to baicalin (25, 50, 100 mg/kg), the mRNA and protein levels of lysozyme in the infection group showed no significant difference (*p* > 0.05), suggesting that baicalin has no effect on the expression of lysozyme [90]. The study also found that the bacterial count in neutrophils of the *S. aureus* infection group was 2.2 × 10^8^ CFU/mL, and after baicalin intervention, the bacterial count in neutrophils decreased in a dose-dependent fashion, with the 25 μg/mL, 50 μg/mL, and 100 μg/mL dose groups reducing to 1.2 × 10^6^ CFU/mL, 2.4 × 10^5^ CFU/mL, and 1.3 × 10^4^ CFU/mL, respectively [90]. Additionally, the study confirmed that baicalin enhances the activity of lysozyme by directly binding to it [90].

## 4. The Solubilization Strategy of Baicalin

**Figure 4:**
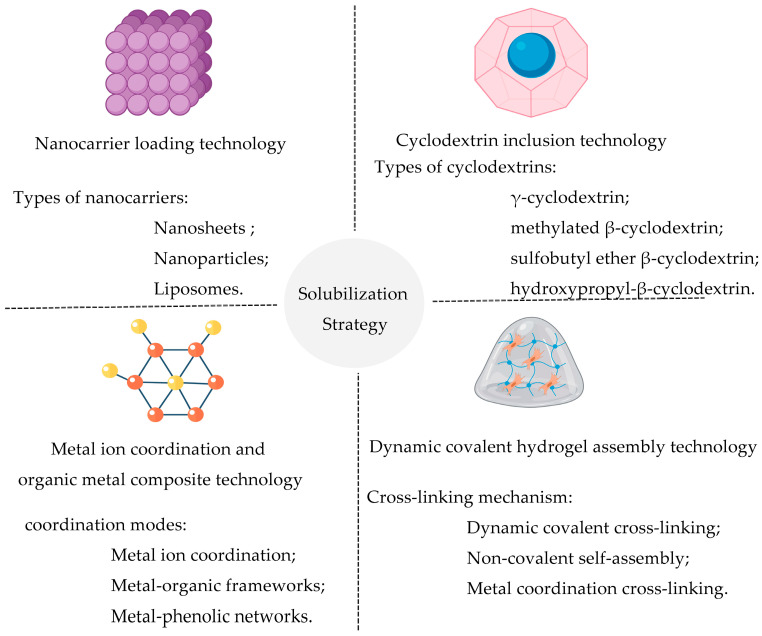
Solubilization strategies for baicalin. Currently, four major solubilization techniques are mainly employed: ① Nanocarrier loading technology (carrier types include nanosheets, nanoparticles, liposomes, etc.); ② Cyclodextrin inclusion technology (common cyclodextrin types include γ-cyclodextrin, methylated β-cyclodextrin, sulfobutyl ether β-cyclodextrin, hydroxypropyl-β-cyclodextrin, etc.); ③ Metal ion coordination and organic–metal composite technology (coordination modes include metal ion coordination, metal–organic frameworks, metal-porphyrin networks, etc.); ④ Dynamic covalent hydrogel assembly technology (crosslinking mechanisms include dynamic covalent crosslinking, non-covalent self-assembly, metal coordination crosslinking, etc.). Each technique enhances the water solubility and bioavailability of baicalin through different molecular/supramolecular interactions.

Despite baicalin exhibiting significant biological activity and application potential in antibacterial and other fields, its inherent physicochemical properties—poor water solubility and low bioavailability—have severely limited its promotion and application in practical scenarios such as clinical treatment and feed additives [91]. To overcome this bottleneck, it is imperative to improve its physicochemical properties and in vivo transport efficiency through targeted solubilization and delivery strategies. Currently developed core technologies mainly include nanocarrier loading technology [92], cyclodextrin inclusion technology [93], metal ion coordination and organometallic complexation technology [94], and dynamic covalent hydrogel assembly technology (Figure 4, Table 2) [95].

### 4.1. Nanocarrier Loading Technology

Nanotechnology has been widely applied in diagnosis, disease treatment, regenerative medicine, gene therapy, dentistry, oncology, the beauty industry, and drug delivery and therapy [96]. The development of nanoscale materials has driven the research and development of novel, highly efficient antibacterial agents, such as silver nanoparticles (NPs) [97] and zinc oxide nanoparticles [98]. In the published related studies, the antibacterial activity of these nanoparticles (e.g., ZnO NPs) has been confirmed. This property can act on various pathogenic bacteria such as *E. coli*, *P. aeruginosa*, and *S. aureus* [97,99]. Furthermore, nanomaterials can serve as nanocarriers for delivering bioactive compounds and therapeutic agents, thereby enhancing drug stability and efficacy [100]. To date, various baicalin-loaded nanocarriers have been constructed and applied, significantly improving the bioavailability and therapeutic effects of baicalin; for instance, Langarudy et al. [101] prepared baicalin-coated zinc oxide nanoparticles (ZnO-Baicalin NPs) and evaluated their antibacterial activity. It was found that ZnO-Baicalin NPs exhibited a stronger inhibitory effect on the biofilm of *S. aureus* compared to ZnO NPs, with the inhibition rate increasing by 17–27% [101]. ZnO-Baicalin NPs also significantly reduced the metabolic activity of *S. aureus* biofilm cells and the expression levels of the quorum sensing genes (*agrA* and *agrD*) [101]. This indicates that the ZnO-Baicalin NPs have an anti-biofilm effect on *S. aureus*.

MXene is an emerging family of two-dimensional (2D) materials, among which Ti_3_C_2_T_X_ MXene exhibits excellent biocompatibility and broad-spectrum antibacterial activity [102]. In a study by Zeng et al. [12], Ti_3_C_2_T_X_ MXene was exfoliated to obtain Ti_3_C_2_T_X_ nanosheets, which were then mixed with baicalin-containing plant extracts and incorporated into a polycaprolactone (PCL) matrix via electrospinning to fabricate PCL hybrid nanofiber membranes loaded with Ti_3_C_2_T_X_ and baicalin. The results revealed that the incorporation of Ti_3_C_2_T_X_ nanosheets and baicalin significantly improved the hydrophilicity of the membranes, facilitating the sustained release of baicalin [12]. Antibacterial assays demonstrated a strong synergistic effect between Ti_3_C_2_T_X_ nanosheets and baicalin, enabling the PCL hybrid nanofiber membranes to exhibit excellent antibacterial activity against *S. aureus*. Notably, the PCL-4 membrane (containing 5 wt% baicalin + 3 wt% MXene) nearly completely suppressed the growth of *S. aureus* after 120 h [12].

Nanolipid delivery systems offer a feasible solution to the low oral bioavailability of poorly water-soluble bioactive substances such as baicalin. Wu et al. [103] successfully developed a novel baicalin nanoemulsion, which showed significantly enhanced intestinal absorption efficiency. Specifically, the absorption rate constant (Ka) of the nanoemulsion in the duodenum, jejunum, ileum, and colon was 10.75-fold, 8.19-fold, 3.94-fold, and 1.66-fold higher than that of baicalin suspension, respectively, with an average 6.14-fold increase in Ka throughout the full length of the intestinal tract [103]. Compared to baicalin aqueous suspension, the nanoemulsion increased the oral exposure (AUC_0-t_) of baicalin by 14.56-fold, the peak concentration (C_max_) by 3.39-fold, and the elimination half-life by 3.72-fold [103]. These improvements significantly prolonged the drug’s retention time in vivo and reduced its clearance rate [103]. Additionally, Lu et al. [104] found that polyvinyl alcohol-chitosan electrospun nanofiber membranes loaded with baicalin liposomes could inhibit mushroom weight loss, browning, spoilage, and bacterial growth, with colony inhibition rates of 79.05% against *E. coli* and 71.12% against *S. aureus*.

### 4.2. Cyclodextrin Inclusion Technology

Baicalin exhibits relatively low bioavailability, which presents significant challenges for its clinical application as an antibacterial agent. Thus, a formulation strategy is required to enhance baicalin’s solubility in physiological environments while improving its permeability across biological membranes. Cyclodextrin, as a superior carrier, can effectively enhance the water solubility of lipophilic drugs and improve their bioavailability [105,106,107], can increase a drug’s solubility and permeability in the unstirred aqueous layer adjacent to the outer surface of cell membranes [108,109]. Additionally, cyclodextrins interact with cell membrane components by extracting phospholipids and cholesterol, thereby enhancing the membrane’s permeability to the drug [110].

Jakab et al. [111] compared six different baicalin-cyclodextrin (CD) inclusion complexes and found that, relative to baicalin’s solubility in distilled water, the solubility of baicalin increased by 5.47-fold, 2.88-fold, 2.55-fold, and 1.59-fold after inclusion with γ-cyclodextrin, randomly methylated β-cyclodextrin, sulfobutyl ether β-cyclodextrin, and hydroxypropyl-β-cyclodextrin, respectively. In contrast, α-cyclodextrin and β-cyclodextrin did not exert significant solubilizing effects when complexed with baicalin. These results indicate that γ-cyclodextrin is the optimal carrier for improving baicalin’s solubility [111]. Paladini et al. [112] developed a soluble baicalin formulation by complexing baicalin with sulfobutyl ether-β-cyclodextrin. This host–guest inclusion complex significantly increased the solubility of baicalin (110-fold higher than free baicalin) and exhibited markedly superior antibiofilm activity compared to free baicalin. Notably, to achieve equivalent antibacterial effects, this inclusion complex required only 1/8 to 1/9 the concentration of free baicalin [112]. In another study, baicalin can enter the hydrophobic cavity of hydroxypropyl-β-cyclodextrin through supramolecular assembly, thereby forming a stable inclusion complex. This modification increased baicalin’s water solubility from 0.0170 mg/mL to 8.9600 mg/mL. Compared to unmodified baicalin, this inclusion complex demonstrated excellent antibacterial activity against *E. coli*, *S. aureus*, and *C. albicans* [42].

### 4.3. Metal Ion Coordination and Organometallic Composite Technology

Many drug molecules—including flavonoids and antibiotics—exhibit limited aqueous solubility, primarily attributable to their high hydrophobic character arising from extensive nonpolar structural motifs. To address this limitation, metal ions such as Zn^2+^, Cu^2+^, and Co^2+^ serve as positively charged central ions capable of forming coordination bonds with electron-donating functional groups in drug molecules—particularly carboxyl (-COOH), phenolic hydroxyl (-OH), and carbonyl (C=O) moieties—thereby increasing molecular polarity and improving physicochemical properties [113]. Baicalin, for instance, features an extended π-conjugated aromatic scaffold and multiple electron-rich coordinating sites, including ortho-dihydroxyphenyl (catechol), carboxyl, and carbonyl groups, rendering it highly competent for stable chelation with transition metal ions. Liu et al. [114] synthesized baicalin–manganese complexes (BCM) and systematically evaluated their antibacterial efficacy and mechanism of action. Results demonstrated that both the 1:1 and 2:1 (baicalin:Mn^2+^) BCM formulations exhibited significantly enhanced antibacterial activity relative to uncomplexed baicalin [114]. Notably, the 1:1 BCM complex displayed an eightfold increase in potency against *S. aureus* compared with native baicalin. Crucially, serial passage of *S. aureus* over 20 generations in subinhibitory concentrations of conventional antibiotics (amikacin, azithromycin, clindamycin) led to marked resistance development—evidenced by 32-fold, 8-fold, and 4-fold increases in minimum inhibitory concentration (MIC), respectively—whereas the MIC of BCM remained unchanged across all passages [114]. Furthermore, BCM effectively reversed pre-existing resistance to amikacin and azithromycin in resistant *S. aureus* strains, underscoring its potential to circumvent or suppress antimicrobial resistance [114]. Collectively, these findings indicate that baicalin-manganese coordination complexes combine potent, broad-spectrum antibacterial activity with a low propensity for resistance induction.

Metal–phenolic networks (MPNs) constitute a class of dynamic, supramolecular organic-inorganic hybrid materials constructed via reversible coordination-driven assembly between multivalent metal ions and polyphenols; especially catechol- or pyrogallol-containing compounds [115]. Owing to their rapid surface-adaptive assembly and capacity to form robust, three-dimensional crosslinked architectures, MPNs enable versatile nanoscale platform engineering on diverse substrates [116]. The choice of metal ion critically governs network stability, redox activity, biodegradability, and functional performance-thus enabling rational design of application-specific materials [117]. Zhang et al. [118] developed berberine–baicalin-based nanoparticles (BA-BBR@MPN NPs) through co-assembly of berberine (BBR) and baicalin (BA), subsequently modified with caffeic acid and Fe^3+^ to construct a metal–phenolic coating. Comprehensive in vitro and in vivo evaluations validated that these caffeic acid-Fe^3+^-coated, dual-drug-loaded nanoparticles exhibit broad-spectrum antibacterial activity, while maintaining excellent biocompatibility and demonstrating pronounced pro-healing effects in wound models [118]. In parallel, Huang et al. [119] engineered a folate-functionalized, macrophage-targeted metal–organic framework (MOF), designated BA@FA-UiO-66-NH_2_, for selective delivery of baicalin (BA) to M1-polarized macrophages. This system achieves high drug loading efficiency and superior biocompatibility, and synergistically alleviates osteoarthritis pathology by concurrently modulating macrophage polarization and scavenging reactive oxygen species (ROS) [119].

### 4.4. Dynamic Covalent Hydrogel Assembly Technology

The research and application of various chrysophanol-based hydrogels have effectively improved the aqueous solubility of chrysophanol and significantly overcome the technical bottlenecks in its clinical translation and practical application. As a class of functional materials with considerable application potential, responsive hydrogels can specifically respond to multiple external stimuli, including pH, temperature, ionic concentration, redox reagents, enzymes, and ultraviolet radiation, thereby having great prospects in various fields of biomedicine, such as drug delivery, regenerative engineering, and biosensing [120,121]. Hydrogels are three-dimensional polymeric network structures formed via chemical crosslinking agents through covalent or non-covalent bonds, and can be fabricated into diverse spatial morphologies such as nanofibers, nanotubes, and nanocapsules [122]. The responsive properties of these materials rely on precise structural design; core strategies primarily involve incorporating polymers with responsive covalent bonds (e.g., disulfide bonds, borate ester bonds) or utilizing supramolecular interactions (e.g., host–guest recognition) to achieve stimulus-responsive regulation.

Recently, Wang et al. [123] developed a dynamic covalent network hydrogel composed solely of chrysophanol and inorganic borate salts. In their study, the chrysophanol hydrogel group exhibited a remarkably larger inhibition zone compared to the free chrysophanol group, indicating enhanced antibacterial activity. Fluorescence staining experiments further confirmed this finding: the chrysophanol hydrogel group showed a marked increase in red fluorescence (indicating dead bacteria), reflecting a significant reduction in viable bacterial counts. Notably, this dynamic covalent hydrogel also demonstrated excellent biocompatibility [123]. In another study, Wang et al. [124] prepared a carrier-free binary metal–organic small-molecule hydrogel (BA-Zn (II) hydrogel). By leveraging the synergistic effect between chrysophanol and Zn^2+^, this hydrogel addressed the weak antibacterial activity of chrysophanol alone [124]. At the same concentration, the antibacterial rate of the BA-Zn (II) hydrogel reached 90.31 ± 0.34%, more than five times that of chrysophanol alone, with a significant decrease in bacterial survival [124]. Plate counting assays revealed that at a concentration of 250 μM, the number of bacterial colonies in the BA-Zn (II) hydrogel group was substantially lower than that in the chrysophanol monotherapy group [124]. Morphologically, bacteria treated with the BA-Zn (II) hydrogel exhibited apparent shrinkage, rupture, and disrupted cell wall integrity, whereas bacteria treated with chrysophanol alone exhibited no significant morphological differences from the control group (maintaining a smooth spherical shape) [124]. Collectively, the BA-Zn (II) hydrogel exhibits superior antibacterial efficiency, bactericidal capacity, and bacterial structure-destructive effects, while eliminating the need for additional carriers, thus ensuring higher biocompatibility [124].

Furthermore, a carrier-free binary small-molecule hydrogel (BA-SAN) was successfully fabricated via the direct self-assembly of baicalin (BA) and sanguinarine (SAN), wherein the network formation is stabilized by non-covalent forces such as electrostatic attraction, π-π stacking, and hydrogen bonding [125]. Owing to their plasticity and injectability, these hydrogels are often used as wound dressings. Wang et al. [125] reported that the BA-SAN hydrogel exhibits antibacterial activity against MRSA. In the SAN group and BA-SAN hydrogel group, an increased transcriptional level was observed for the *lytH* gene, while the mRNA expression of the differentially expressed genes such as *pflA*, *fib*, *hld*, *agrB*, and *agrD* exhibited the opposite trend and was significantly reduced [125]. Additionally, the BA-SAN hydrogel group showed reduced mRNA expression of the *hlyII* and *eta* genes. These genes play vital roles in modulating bacterial nucleotide metabolism, protein synthesis, ribosome biogenesis, and the generation of virulence factors. For instance, the *lytH* gene may encode a cell wall acylase, and its deficiency increases MRSA resistance. Upregulated *lytH* expression following treatment with SAN or BA-SAN hydrogel may contribute to reduced MRSA resistance. Moreover, the BA-SAN hydrogel can inhibit the expression of genes correlated with the MRSA agr system, suggesting its potential to inhibit the synthesis of virulence factors associated with skin infections [125]. Mankar et al. [126] also developed a Soluplus–polyvinyl alcohol (PVA) composite hydrogel patch loaded with baicalin for wound healing promotion. This hydrogel patch effectively improved baicalin solubility, achieving a transdermal delivery efficiency of 93.24% at 12 h [126]. Additionally, it exhibited a Draize score of 0, indicating excellent biocompatibility and no skin irritation [126]. In rat models, treatment with this patch resulted in a wound contraction rate that was 10.4% higher than that of standard treatment and 34.9% higher than that of the control group. Histologically, the patch promoted accelerated epithelial regeneration and granulation tissue repair [126].

## 5. Conclusions and Outlook

To address the challenge of bacterial resistance to traditional antibiotics, extracting antibacterial active components from natural plants has emerged as a promising and viable strategy. Baicalin, a flavonoid compound primarily isolated from the dried roots of *Scutellaria baicalensis*, is a key component of traditional Chinese medicine (TCM). Owing to its abundant natural sources, diverse biological activities, and continuous advancements in drug delivery technologies, baicalin has become increasingly prominent in modern scientific research.

This review summarizes the biological characteristics of baicalin, including its selectivity for toxicity, stability, low drug resistance, pharmacokinetics, and specificity. It also discusses the multiple antibacterial mechanisms of baicalin, including inhibiting bacterial adhesion, disrupting biofilm formation, inhibiting the gene of the efflux pump, and interfering with quorum sensing and virulence pathways—all of which collectively reduce bacterial load and mitigate the tissue damage it causes. Additionally, baicalin can alleviate bacteria-induced inflammatory responses by attenuating the expression of inflammatory factors through various pathways; for example, regulating microRNA expression, inhibiting inflammatory signaling cascades, and modulating the gut microbiota. In terms of immune regulation, baicalin mitigates bacteria-induced immunosuppression and enhances antibacterial immune effects by directly regulating the quantity and function of immune cells, targeting key immune signaling pathways, modulating cytokine secretion, and activating autophagic pathways. However, most current studies on the mechanisms of action of baicalin have been conducted in vitro or animal models, and its clinical efficacy and safety require further in-depth investigation. Therefore, well-designed clinical trials are necessary to systematically evaluate the safety, pharmacokinetics, and therapeutic efficacy of baicalin. Furthermore, the poor water solubility and low bioavailability of baicalin have limited its clinical translation and application. To overcome these inherent limitations, various solubilization techniques have been developed and applied. For instance, encapsulating baicalin in nanocarriers, complexing it with cyclodextrins, chelating it with metal ions, or formulating it into hydrogels can effectively improve the solubility, stability, and bioavailability of baicalin, thereby enhancing its therapeutic potential. Despite these advancements, existing solubilization systems still have inherent shortcomings: some inclusion complexes exhibit low drug loading and limited solubilization efficiency; nanocarrier systems are associated with complex preparation processes, high production costs, and poor in vivo stability; and certain composite carriers fail to achieve targeted delivery of baicalin and may pose potential risks, such as carrier material cytotoxicity and poor biodegradability. Thus, the focus of future research on solubilization techniques should be two-fold. On the one hand, it is essential to optimize existing solubilization processes—for example, using response surface methodology and orthogonal experiments to optimize parameters such as the inclusion ratio, preparation temperature, and stirring speed—thereby improving the drug loading capacity, solubilization ratio, and stability of solubilization systems. On the other hand, efforts should be directed toward developing novel solubilization carriers and technologies, combined with molecular simulation techniques, to precisely screen optimal carrier materials with excellent biocompatibility and biodegradability. In addition, exploring the combined application of multiple solubilization techniques will help to compensate for the deficiencies of individual solubilization methods, further enhancing the solubility and bioavailability of baicalin and facilitating its clinical application and industrialization.

## Figures and Tables

**Table 1:** Selective toxic effects of baicalin in different models.

Research Model	Administration Route	Dose/ Treatment	Toxicity Indicators	Safety Evaluation	References
MDA-MB-231 cells, MCF-7 cells, RAW 264.7 cells, hMSC cells, murine pre-osteoblasts/MC3T3-E1 cells	In vitro	1 nM, 10 nM, 100 nM.	1. The vitality of breast cancer cells decreases. 2. The differentiation of osteoclasts is inhibited. 3. The expression of osteogenesis-related genes is downregulated. 4. The mitochondrial pathway is activated.	1. It has no obvious toxicity to normal bone cells (hMSC, osteoblasts). 2. It selectively kills breast cancer cells while being safe for normal bone marrow/bone-derived cells. 3. It takes effect at low concentrations (nanomolar level) without significant toxicity to organs.	[14]
Human liver cancer cells (MHCC-97H, HCC-LM3), human normal liver cells (QSG-7701), mouse primary liver cells	In vitro	0.1, 1, 10, 20, 40, 80, 160, 320, 640 μM.	Cell viability (CCK-8)	1. It has inhibitory effects on MHCC-97H and HCC-LM3. 2. It has less cytotoxicity on QSG-7701 and mouse primary liver cells. 3. At 24 h, the IC50 of liver cancer cells is significantly lower than that of normal liver cells, showing selective killing of tumors.	[15]
PDX model derived from patients with renal cancer	Intraperitoneal injection (baicalin); Gavage (sunitinib)	100 mg/kg/d, administered continuously for 6 weeks.	1. Cardiac toxicity is evaluated by myocardial injury, myocardial cell apoptosis, and myocardial fibrosis. 2. Serum biochemistry is assessed via creatine kinase (CK) and lactate dehydrogenase (LDH). 3. Cardiac histology is examined using H&E staining, Tunel staining, and Masson staining. 4. Cardiac index is calculated as the ratio of heart weight to body weight.	1. Using scutellarein alone has no obvious cardiac toxicity and does not affect the body weight of mice. 2. When combined with sunitinib, it can reduce the myocardial injury, apoptosis, and fibrosis induced by sunitinib, reverse the increase in CK and LDH and the abnormal HW/BW. 3. It has no significant adverse effect on liver and kidney function-related biochemical indicators (TP, AST, ALT, ALP, Crea, Urea, GLU). 4. It does not affect the anti-tumor effect of sunitinib, and the tumor inhibitory effect remains significant after combination use.	[16]
Normal human liver cell line L02	In vitro	0–200 μg/mL	1. Cell viability is measured by the CCK-8 assay. 2. Apoptosis rate is detected using Annexin V-FITC/PI staining. 3. S-phase arrest of the cell cycle is evaluated. 4. Apoptotic proteins (Bax, Bcl-2) are analyzed. 5. Signal proteins (p-JNK, JNK, p-c-Jun, c-Jun) are examined.	1. At concentrations ranging from 0 to 100 μg/mL, it shows no significant toxicity to normal L02 liver cells and does not inhibit cell survival. 2. It can alleviate AFB1-induced liver toxicity and cell apoptosis. 3. It inhibits the JNK/c-Jun pathway, reduces pro-apoptotic proteins, and increases anti-apoptotic proteins. 4. It is safe for normal liver cells and has a clear liver-protective effect.	[17]
Human embryonic lung fibroblast cell line WI-38; LPS-induced acute lung injury (ALI) cell model	In vitro	25 M	1. Cytotoxicity is evaluated by cell viability (MTT assay) and proliferation ability (EdU assay). 2. Apoptosis is assessed via flow cytometric apoptosis rate, Caspase3 activity, and TUNEL positive rate. 3. Oxidative damage is measured by MDA and ROS levels. 4. Inflammatory damage is determined based on TNF-α, IL-6, and IL-1β secretion levels.	1. Baicalin itself has no obvious cytotoxicity and does not inhibit the growth of normal lung cells. 2. It can significantly reduce LPS-induced apoptosis, oxidative stress and inflammatory responses in lung cells. 3. It does not cause additional cell damage and is safe for WI-38 cells. 4. It exerts protective effects by regulating the METTL14/SOX6 axis, without activating toxic pathways.	[18]
Canine renal tubular epithelial cells (MDCK); cisplatin (20 μmol/L) induced cytotoxic injury model	In vitro	Safe dose: ≤ 50 μmol/L; Effective protective dose: 25 μmol/L, 50 μmol/L.	1. Cytotoxicity is evaluated by cell viability (CCK-8 assay) and live/dead staining (Calcein/PI). 2. Apoptosis is assessed via flow cytometric apoptosis rate, BAX/BCL-2 ratio, and p53 protein expression. 3. Oxidative damage is measured by MDA, SOD, CAT, and GSH levels. 4. Inflammatory damage is determined based on TNF-α, IL-1β, IL-6, and NO levels. 5. Autophagy is analyzed by the expression of Beclin1, P62, p-AMPK, and p-mTOR.	1. Baicalin at a concentration of ≤50 μmol/L has no direct toxicity to MDCK cells and does not inhibit the growth of normal cells. 2. It can significantly reduce cisplatin-induced apoptosis, oxidative stress, and inflammatory response. 3. It dose-dependently improves cisplatin-induced inhibition of autophagy function. 4. The overall safety is good, and it causes no obvious direct damage to liver and kidney cells.	[19]
Human immortalized keratinocyte cells (HaCaT); UVC induces cell damage model	In vitro	25, 50, 100, 150, 200 μg/mL.	1. Cytotoxicity is evaluated by MTT cell viability assay. 2. Apoptosis is assessed via Sub-G1 phase apoptosis rate. 3. Oxidative damage is measured by ROS level. 4. DNA damage is determined by cyclic pyrimidine dimers and oxidative adducts.	1. When used alone at concentrations ≤ 200 μg/mL, baicalin is non-toxic to HaCaT cells and does not induce apoptosis, ROS production or DNA damage. 2. It dose-dependently reduces UVC-induced cell death, apoptosis, ROS generation, and DNA damage. 3. It does not affect the cell cycle.	[20]

**Table 2:** Strengths and limitations of diverse solubilization strategies for baicalin.

Solubilization Technology	Strengths	Limitations	References
Nanocarrier loading technology	1. Enhances drug stability and bioavailability. 2. Exerts a synergistic antibacterial effect with prominent activity. 3. Shows good biocompatibility and promotes wound healing. 4. Has a wide application scope.	1. Involves a complex preparation process (e.g., nanomaterial preparation, electrospinning). 2. Has poor universality, requiring targeted research on nanocarrier adaptability.	[12,96,97,98,99,100,101,102,103,104]
Cyclodextrin inclusion technology	1. Significantly improves water solubility and bioavailability. 2. Enhances the permeability of the cell membrane to the drug. 3. Exhibits superior antibacterial membrane activity compared to free chrysophanic acid, and requires a lower drug concentration for the same antibacterial effect.	1. The choice of cyclodextrin type has a significant impact on the effect, and it is necessary to select the appropriate cyclodextrin. 2. The inclusion process may be affected by multiple factors, making process optimization quite challenging.	[42,105,106,107,108,109,110,111,112]
Metal ion coordination and organic–metal composite technology	1. Has a broad antibacterial spectrum and significant antibacterial activity. 2. Acts through diverse antibacterial mechanisms, with a low likelihood of developing drug resistance. 3. Is capable of reversing the antibiotic resistance of bacteria in some cases. 4. Involves a relatively simple preparation process.	1. The selection of metal ions should be tailored to specific applications. 2. Some metal ions may pose certain toxicity risks, so the dosage must be strictly controlled and low-toxicity metal ions should be chosen.	[113,114,115,116,117,118,119]
Dynamic covalent hydrogel assembly technology	1. Enhances solubility and breaks through the limitations of traditional hydrogels. 2. Some formulations do not require additional carriers and have higher biological safety. 3. Possesses good degradability, multi-stimulus responsiveness, plasticity, and injectability. 4. Can inhibit the generation of bacterial virulence factors, alleviate inflammatory responses, and promote wound healing.	1. The loading capacity of traditional hydrogels is relatively low. 2. Some hydrogels rely on specific non-covalent bonds for self-assembly, and the assembly conditions are rather demanding.	[120,121,122,123,124,125,126]

## Data Availability

No new data were created or analyzed in this study. Data sharing is not applicable.

## Abbreviations

AIP, autoinducing peptide; APEC, avian pathogenic *Escherichia coli*; ARGs, antibiotic resistance genes; BA, baicalin; BBR, berberine; BCM, baicalin–manganese complexes; CD, cyclodextrin; DCs, dendritic cells; ExPEC, extraintestinal pathogenic *Escherichia coli*; FFAR2, free fatty acid receptor 2; IκBα, nuclear factor κB inhibitor α; IL-6,interleukin-6; IL-8, interleukin-8; IL-10, interleukin-10; JNK, c-Jun N-terminal kinase; LPS, lipopolysaccharide; MDR, multidrug-resistant; MIC, minimum inhibitory concentration; MOF, metal–organic framework; MPNs, Metal–phenolic networks; MRSA, methicillin-resistant *Staphylococcus aureus*; NF-κB, Nuclear factor kappa-light-chain-enhancer of activated B cells; NLRP3, nucleotide-binding oligomerization domain-like receptor family pyrin domain-containing 3; NPs, nanoparticles; p38 MAPK, p38 mitogen-activated protein kinase; PCL, polycaprolactone; PVA, polyvinyl alcohol; PVL, Panton-Valentine leukocidin; QS, quorum sensing; rPVL, recombinant PVL; SAN, sanguinarine; SBE-β-CD, sulfobutyl ether-β-cyclodextrin; SCFA, short-chain fatty acid; SrtB, Sortase B; sub-MIC, sub-minimum inhibitory concentration; TCM, traditional Chinese medicine; TCS, two-component system; TLR, Toll-like receptor; TNF-α, tumor necrosis factor-α; 2D, two-dimensional.
